# Comparison between pulse pressure variation and conventional parameters as guides to resuscitation in a pig model of acute hemorrhagic shock with endotoxemia

**DOI:** 10.1186/cc9470

**Published:** 2011-03-11

**Authors:** J Noel-Morgan, DT Fantoni, DA Otsuki, JO Auler

**Affiliations:** 1 Faculdade de Medicina da Universidade de São Paulo, Brazil

## Introduction

Volume expansion is often used in anesthesia and critical care to improve oxygen delivery and, in mechanically ventilated patients, pulse pressure variation (PPV) has been proposed as an index to aid in the assessment of the appropriate amount of fluids to be administered to this end [1]. The objective of this study was to compare PPV with conventional parameters as guides to resuscitation, in an experimental model of severe hemorrhagic shock with endotoxemia.

## Methods

Twenty-seven anesthetized, mechanically ventilated pigs were submitted to acute hemorrhagic shock with infusion of endotoxin. Animals were randomly allocated to three groups: control (*n *= 9); conventional treatment with lactated Ringer's (LR) to achieve and maintain central venous pressure (CVP) ≥12 mmHg, mean arterial pressure (MAP) ≥65 mmHg and SvO_2 _≥65% (CNV, *n *= 9); or LR to achieve and maintain PPV ≤13% and MAP ≥65 mmHg (dPP, *n *= 9). Hemodynamic parameters, measured by pulmonary artery catheter and femoral arterial catheter, and blood gases were assessed at baseline (TB), 1 hour after hemorrhage (TS), and hourly during the treatment period (T1 to T3). Groups and times were compared with two-way ANOVA followed by Tukey test and *t *test was used for comparisons of treatment times and LR amounts (*P *< 0.05).

## Results

At TS all groups presented equivalent, significant decreases in cardiac index (CI), MAP, CVP, SvO_2 _and oxygen delivery index (DO_2_I) and an increase in PPV (all *P *< 0.001). At T3, both treated groups presented hemodynamic recovery, with no statistical difference from TB or each other for CI, MAP, SvO_2_, DO_2_I or PPV. Statistically, there were no differences in times or amounts of LR to achieve endpoints, for maintenance or in total amounts of LR given. The only statistical difference between treatment groups involved CVP, which was higher in group CNV than in group dPP at T2 (*P *= 0.009) and T3 (*P *< 0.001). CVP was also higher at T3, in group CNV, when compared with TB (*P *= 0.006).

## Conclusions

Although early fluid management guided by PPV yielded similar hemodynamic results to those achieved by management through conventional parameters, a difference could be noted regarding CVP, which was maintained higher in group CNV, but was restored to baseline values by PPV-guided therapy. The clinical impacts of such occurrences remain to be determined.
